# For whom and under what circumstances do school-based energy balance behavior interventions work? Systematic review on moderators

**DOI:** 10.3109/17477166.2011.566440

**Published:** 2011-06-09

**Authors:** Mine Yildirim, Maartje M Van Stralen, Mai J M Chinapaw, Johannes Brug, Willem Van Mechelen, Jos W R Twisk, Saskia J Te Velde

**Affiliations:** 1 Department of Public and Occupational Health and the EMGO Institute for Health and Care Research, VU University Medical Center, Amsterdam, the Netherlands; 2 Department of Epidemiology and Biostatistics and the EMGO Institute for Health and Care Research, VU University Medical Center, Amsterdam, the Netherlands

**Keywords:** Children, diet, intervention, moderator, overweight, physical activity

## Abstract

The aim of this review was to systematically review the results and quality of studies investigating the moderators of school-based interventions aimed at energy balance-related behaviors. We systematically searched the electronic databases of Pubmed, EMBASE, Cochrane, PsycInfo, ERIC and Sportdiscus. In total 61 articles were included. Gender, ethnicity, age, baseline values of outcomes, initial weight status and socioeconomic status were the most frequently studied potential moderators. The moderator with the most convincing evidence was gender. School-based interventions appear to work better for girls than for boys. Due to the inconsistent results, many studies reporting non-significant moderating effects, and the moderate methodological quality of most studies, no further consistent results were found. Consequently, there is lack of insight into what interventions work for whom. Future studies should apply stronger methodology to test moderating effects of important potential target group segmentations.

## Introduction

Childhood obesity has risen in both developed and developing countries among both genders and among all ethnic, and socioeconomic groups (1,2). Lower socioeconomic groups and certain ethnic minorities have been hit hardest (3). Obesity is the result of a long-term positive energy balance, i.e., when energy intake is larger than energy expenditure. Intake and expenditure are influenced by so-called energy balance-related behaviors (EBRBs) (4); i.e., specific dietary, physical activity (PA) and sedentary behaviors. The importance of effective interventions that aimed at improving EBRBs in order to prevent obesity in youth was highlighted in previous literature reviews (5,6).

Obesity risk may differ and interventions may not be equally effective across subgroups, such as those based on socioeconomic status, ethnicity or race, age, and gender (6). One identical intervention strategy (a one-size-fits-all intervention) may not cover the diverse needs of various subgroups; e.g., some may need different types or doses; highly motivated ones may need not more than encouragement (7). Intervention developers can benefit from considering moderating effects by tailoring the intervention content to specific subgroups (8).

Exploring ‘for whom’ or ‘under what circumstances’ interventions work or not is possible with moderation analysis. Moderators are variables that affect the direction and/or strength of the relation between independent and outcome variables (9,10). Examples are factors that are manipulated by the intervention (e.g., family involvement), situational (e.g., the site or setting where the intervention is conducted), socio-demographic (e.g., gender), or psychological variables (e.g., motivation towards behavioural change at the start of the intervention) (10,11).

A moderating effect also called effect modification can be tested by including an interaction term created by multiplying the moderator and the independent variable into the analysis. Conducting subgroup analyses without a previous test of interaction is not advisable, as repeated statistical testing on the same dependent variable for each subgroup increases the risk of obtaining a false positive result (12,13). In case of significant moderation, complementary exploratory analyses within subgroups according to the moderator are needed (14).

To date, only two systematic reviews on moderators of obesity prevention interventions among children have been published. Kremers et al. (15), investigated moderators of intervention effects on EBRBs including only so-called environmental interventions. Stice et al. (16) included studies investigating moderators of obesity intervention effects on overweight indicators. Both reviews found moderating effects of age, gender, and race, but were based on small numbers of studies (15) or lacked studies with long-term follow-up (16). Neither of the reviews performed quality assessments of the included studies or the moderation analyses applied.

The aims of this systematic review were to identify the most important moderators, and to summarize and assess the quality of studies investigating moderators of school-based interventions aimed at EBRBs among school-aged children. We conclude with suggestions for future school-based obesity prevention interventions.

## Methods

### Literature search

We identified relevant articles through systematic searches in the electronic databases of Pubmed, EMBASE, Cochrane, PsycInfo, ERIC and Sportdiscus. Searches were limited to studies among humans, written in English and published between January 1990 and October 2009. The search terms were based on Boolean logic and included AND-combinations between terms standing for children and adolescents, for school-based intervention and for EBRBs. Since studies are often not framed as a moderation test and generally do not include related terms in their keywords or abstract section, moderator terms were not included in the search strategy. To eliminate an excess number of articles in our broad literature search, NOT-combinations were used for unrelated topics based on our previous experience. The search strategy for the database of Pubmed is shown in online Figure 1.

**Figure 1:**
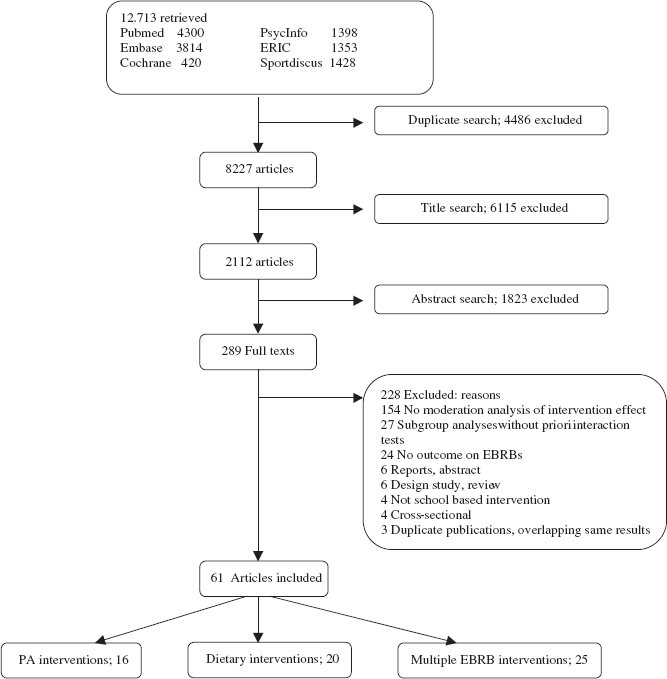
Article search and study selection.

### Inclusion and exclusion criteria

Inclusion criteria were that the study: (i) had to be a randomized controlled trial (RCT) or quasi-experimental controlled studies aimed at primary prevention of overweight; (ii) targeted EBRBs (PA, sedentary or dietary behaviors) in order to prevent overweight or overweight-related diseases; (iii) was conducted among children and/or adolescents aged between 4 and 18 years; and (iv) applied an appropriate test of moderation (i.e., contained a test of an interaction). Moderators that were included were experimentally manipulated, situational, personal or psychosocial variables. Only full text articles were included. Studies that aimed to change preferences, taste, product sale and content of school lunch were excluded. Studies that were not only school-based (combined with home components) were included in this review.

### Process of study selection

Author MY scanned all titles of retrieved studies for relevance. Afterwards, authors MY and MVS independently screened abstracts for possible relevance and decided together on inclusion or exclusion. Next, the same two authors independently checked the full text versions of potentially relevant articles. Authors' differences regarding inclusion were resolved by discussion. A list of excluded studies after full text search and related reasons for exclusion can be obtained from the first author upon request.

### Data extraction and quality assessment

All included studies were evaluated and data were abstracted by two authors (MY, MVS) independently and differences resolved by discussion. A third author (MC) was approached in case of disagreements. For each article, the following data were extracted: (i) study population; (ii) study design; (iii) intervention content; (iv) theory that the study was based on; (v) EBRBs outcome variables and measures; (vi) intervention effect on outcome variable; (vii) moderators tested; (viii) results of the moderation analyses; and (ix) in case of significant moderators, results of the stratified analyses.

The quality of the studies was assessed using the items from a Delphi list (17) and from the checklist for evaluating moderation analysis from Frazier et al. (9). Quality items are indicated in online Table II. Among these items, we evaluated point estimates and measures of variability as appropriate in case mean, standard deviation, standard error, median or quartiles for the primary outcomes were provided. Criteria have a ‘yes’ (+ = 1), ‘no’ (− = 0) or ‘don't know’ (? = 0) answer format. In case of inadequate information in the text, the reviewers contacted the first author of the study. A quality score was calculated for each study by summing up the scores for each individual quality item, resulting in a possible score of 0-11. The studies were graded arbitrarily as being of a relatively low quality when the total quality score was between 0 and 4, as medium between 5 and 8 and being of a relatively high quality when scores were between 9 and 11.

Moderation analysis requires several conditions that were also evaluated in our quality assessment. First, the selection of moderators should be based on a specific rationale - theory or evidence based -explaining why the intervention may be more effective for some subgroups than for others. This should be made prior to the intervention planning stage (9). Second, the statistical power of moderation analysis should be considered. Several factors common in social science research can decrease power (e.g., low sample sizes, low effect sizes), which can lead to an incorrect conclusion of no moderating effect (11). Third, homogeneity of residual variances should be checked for categorical moderators. This assumes that the residual variances, which are the error variances that remain after predicting a dependent variable from the independent variables, remain constant across the moderator categories (11,18).

## Results

Table I shows the flow of the studies through the review process and the reasons for exclusion. In total 12.713 articles were identified. After scanning titles and abstracts, we retrieved 289 articles for full text search. Sixty-one articles met our inclusion criteria (19-79). Twenty dietary interventions, 16 PA interventions and 25 multiple EBRB interventions were included. In studies examining only dietary interventions, the majority (16 out of 20) solely aimed to change fruit and vegetable (FV) intake. Multiple EBRB interventions also aimed to improve dietary intake such as fat, sweets and soft drinks consumption and aimed to change PA and sedentary behavior. Some included studies were based on the same intervention (e.g., Squire's Quest, CATCH, Pathways).

**Table I:** The results of the moderation analyses of the school-based interventions aimed at energy balance-related behaviors.

Moderators	Total PA	Sedentary behavior	Active transport	Fat intake	FV intake	Total energy intake	Snacking/fast food	Sugar/sweets intake	Soft drink consumption
**Personal**									
Gender (female)	0,0,0,0,0,0,0,0,0,0,0,0,0,0,0,0,0,+,+,+,+,+,−	0,0,0,0,0,0,0,−,−	0,0,0,+	0,0,0,0,0,0,0,0,0,+,+,+,+	0,0,0,0,0,0,0,0,0,0,0,0,±,+,+,+,+^b^,x	0,0,0,0,0,0	0,0	0,−	0,≈
Age (younger)	0,0,0,0	0,0		0,0,0,±	0,0,0,0,0,0,0,+,+,−	0	0		
Ethnicity (natives)	0,0,0,0,0,≈^a^	0,0,0,0,0	0	0,0,≈(girls),≈	0,0,0,0,0,0,0,0,−,	0,0,0	0		0
Baseline values of outcome (unfavorable)	0,0,0,0,0,0,+			0,0,+	0,+,+	0,0	+		
Baseline weight status (obese)	0,0,0,0,0,0	+,0,0	0	0,0	0,0	0,0			
SES (low)	0,0,0,0,0,+	0	0	0,0,0	0,0,0,0,+,x,x	0,0	+	0	0
Preferences					0				
Health status (baseline CVD risk [high])				0				+(boys)	
Growth (Increase in heights [low])	0			0		0			
**Situational**									
Recess time	0								
Site (State, province)	0,0	0		0	0,0	0			
Type of education (technical)				+^b^					
Lesson location (indoor)	+VPA								
	−walking								
Teacher speciality (classroom teacher)	+								
School food policy (fruit only policy)					+		0	0	
Compliance (high)	+								
Sports club participation	0	0	0						
Country					+				
**Intervention part**									
Family involvement (high)	0	0			+				
Food type provided (fruit)					0,≈,≈				
**Psychosocial**									
Intention (low)	+								
PBC (low)	+								
Attitude (low)	+								
Normative beliefs	0								

O; NS, +; significant for the group mentioned in brackets, − significant in the opposite direction for the group mentioned in brackets. x; NS after stratified analysis, ± Stratified analysis results are not reported, ≈ Significant for both groups.

a Significant in low income group

b Significant in Natives. VPA, vigorous physical activity.

### Quality assessment

Supplementary Table 2 (available online) shows that 42 studies were of medium and 19 studies were of low quality. There were no high quality studies scoring 9 or higher. The majority of the included studies were RCTs. An intention to treat analysis was applied in 15 studies. Dropout rate was selective in 19 studies, while 26 studies did not report on selectiveness. Twenty-four studies out of the 61 were not explicitly based on a theoretical and/or conceptual framework. Most studies (51 out of 61) used an outcome measure that was checked on its reliability and validity. In most studies, the quality of the conducted moderation analyses was low. Only eight studies provided a rationale for interaction tests, one study checked the assumption of homogeneity of (error) variances across moderator groups. Only one study (43) calculated the power of the moderation analysis and their study had adequate power to detect a moderating effect. We tried to calculate the power using information reported in the articles. However, none of the studies reported sufficient information (i.e. the sample sizes and the predictor-outcome correlations across moderator-based subgroups) for the power calculation program for moderation analysis provided by Aguinis (http://mypage.iu.edu/~haguinis/mmr/index.html).

### Description of interventions

Supplementary Table 3 (available online) shows the study characteristics. The majority of the studies (19 studies) were conducted in the United States of America (USA), followed by the United Kingdom (six studies), Belgium (five studies), Australia and the Netherlands (both four studies), Norway (three studies), Ireland (two studies) and New Zealand, Cyprus, Greece, Sweden, France, Canada (all one study). One international study was conducted in which the Netherlands, Norway and Spain participated. The number of schools included in the studies ranged from 1-96, and participant numbers ranged from 122-5106. Three studies were conducted in girls only. Twenty-two studies out of 61 had more than one follow-up while the others had only one follow-up immediately at the end of intervention. Twelve studies used objective measurement methods for PA, such as pedometers and accelerometers. Others used self-reported PA. Lunch observations were used in seven studies, while others used self-report methods for dietary intake. The reliability and validity values of the self-report instruments were mainly lower than the required minimum value of 0.70, as described by Terwee et al. (80).

### Intervention effect on outcome

Supplementary Table 3 (available online) reports the intervention effects on EBRBs. The EBRBs evaluated in the studies were total daily PA, light, moderate and vigorous PA, sedentary behavior, active commuting, FV intake, fruit juice consumption, soft drink consumption, dietary fat intake, total energy intake, sugar intake, snacking behavior, sweet consumption and fast food consumption. Studies aimed at increasing FV intake (totally 19) all resulted in significant intervention effects except for one study (24). PA interventions also resulted in significant improvements in children's PA levels especially on moderate to vigorous PA, except two studies (22,28) out of 16. The majority of the multiple EBRB interventions were effective in changing at least one behavior. Five out of 25 studies did not show any significant effects.

### Moderators

Supplementary Table 3 (available online) summarizes the results of the moderation analyses conducted in the reviewed studies. The most frequently tested moderating variable was gender (in 46 studies out of 61), followed by ethnicity (in 15 studies), age (in 13 studies), socio-economic status (SES) as indicated by family income and parent's educational level (in 11 studies), baseline value of outcomes (in nine studies) and initial weight status (in eight studies). Other personal moderators were examined each in one study. Psychosocial, situational and experimentally manipulated variables were tested also in a small number of studies.

#### Personal moderators

The moderating effect of gender was tested in 46 studies (totally 79 interaction tests on multiple behaviors) with a significant moderating effect in 17 studies (21 tests). Moderation analyses showed that in general girls responded significantly better than boys to the interventions, except for the interventions aimed at changing sedentary behavior and sugar intake (Table I). Girls particularly appeared to respond better to interventions aimed at decreasing their dietary fat intake. Furthermore, baseline levels of outcome behaviors (e.g., baseline FV consumption) moderated the intervention effects on FV consumption, fat intake, snacking and PA in which the groups with unfavorable baseline values benefitted more from the intervention. Age moderated the intervention effect on FV intake in three studies (out of 14). In two studies younger children and in one study older children responded better. The moderation analyses did not yield many statistically significant and consistent results for ethnicity, initial weight status, SES or health status as potential moderators.

#### Intervention moderators

Family involvement in the intervention was a moderator of the intervention effect on FV consumption with high family involvement showing a larger increase in FV intake (34). Food type provided (fruit vs. vegetables) in the study moderated the intervention effect in two dietary interventions but after stratified analyses, there was no differential intervention effect across subgroups (46,47).

#### Psychosocial moderators

In one PA intervention children who had lower baseline scores on intention, attitude and perceived behavioral control (PBC) responded better to the intervention (44). Normative beliefs did not moderate the intervention effect in this study.

#### Situational moderators

Lesson location (57), teacher specialty (57) and compliance (65) to the guided goal setting were moderators of one PA intervention. School food policy (58) and country (72) were moderators of one FV intervention. Type of education (technical vs. normal schools) moderated the intervention effect on fat intake, in which children from technical school responded better to the intervention (38). Average recess time, site (province, state), sports club participation and region did not moderate any intervention effect.

## Discussion

The objective of this systematic review was to examine for whom and under what circumstances school-based interventions aimed at EBRBs work. Among the included studies, gender was the most frequently examined variable, followed by ethnicity, age, SES, baseline levels of EBRBs and baseline body weight status. Interventions aimed at changing FV intake, PA and fat intake were most often affected by the moderators. We did not find a high level of evidence for any of the potential moderators due to the lack of significant and consistent interaction test results. It may, of course, be that intervention effects are not much different for different subgroups, but a possible reason for the lack of moderation found may also be the lack of methodological quality of the included studies. Drop-out analysis, group similarity at baseline and intention-to-treat analysis were not reported or not conducted in more than half of the studies. It was noted by Baron and Kenny (10) that moderation analysis is often applied when there is an unexpected weak or inconsistent relationship between the predictor and the outcome variable. We found that the studies included in the current review were mainly successful (52 studies out of 61) in changing at least one EBRB but still searched for moderating effects. This finding supports our opinion on performing moderation analysis regardless of whether the intervention showed a significant main effect or not. Furthermore, we investigated the possible influence of the methodological quality of the included studies on the obtained moderation analyses results. We found no relationship between study quality and finding significant moderating effects (and also not for finding main effects). Regarding the merits in the design of the studies, some inferences about potential moderators can be derived.

The most convincing evidence was found for the moderating effect of gender; mainly girls responded significantly better to the interventions than boys. The review from Kremers et al. (15) focused on environmental interventions to change EBRBs and found similar results regarding moderating effects of gender, mainly due to girls' better response to the interventions. One possible explanation for this could be that boys are generally more physically active than girls leaving more room for improvement among girls (81). This finding has been linked to differences in motor skills development, body composition, socialization towards sports and physical activity and freedom to involve to activities independently outside the home (82). One way to explore the factors that prevent the success of a school-based intervention in the full range of student is the assessment of process measures. Although a process evaluation was conducted in nine out of 31 studies in which moderating effects were found, only one study (38) conducted a process evaluation across subgroups. The authors found that girls had read the intervention messages significantly more often than boys and girls found the instructions significantly clearer than boys. These results suggest that the intervention was better implemented among girls. There was no information on differences in validity and reliability of outcome measures between girls and boys in the included studies. Nevertheless, Rangul et al. (83) showed that the reliability of a frequently used self-administered questionnaire (WHO-HBSC) to measure PA was significantly higher in girls compared to boys and that facilitates finding an intervention effect in girls rather than in boys. It may also be the case that girls are more likely to respond in a socially desirable way to self-report questionnaires, such as on activity or fruit and vegetable intake. In future interventions, gender specific underlying mechanisms of behavior change should therefore be considered. For instance, Simen-Kapeu et al. (84) found that girls have fewer active role models, more barriers and less perceived benefits of physical activity compared to boys. Future interventions may consider targeting their intervention strategies more to gender characteristics. For instance, favorable effects of active gaming and organized competitive, team, and high intensity sports and exercises (e.g., football, basketball, team handball, cycling) on PA that are popular among boys, have been shown (85,86). Regarding the dietary interventions, girls have higher concerns of body weight gain and body image when compared to boys (87). This can result in a higher interest towards the intervention increasing the likelihood of an intervention effect. In a qualitative study, it was shown that the key motivating factors for boys in terms of healthy eating were sports and physical performance (88).

The second most common moderator was the baseline level of EBRBs (e.g., baseline FV consumption). These moderators particularly showed their moderating effect in dietary interventions. The subgroups with unfavorable baseline values responded better to the intervention on FV consumption, fat intake and snacking. The results indicated that the more the children needed the intervention, or the more room there was for improvement, the more they appeared to benefit from it. Regression to the mean -the fact that participants with extreme scores at baseline generally regress to the population mean of a group - should be considered and avoided in future studies.

In several papers, the importance of parental involvement in childhood overweight prevention interventions has been argued (89,90). Its moderating effect was tested in one multiple behavior intervention (34) and found as a significant moderator of the intervention effect on FV intake but not on total PA and sedentary behavior. In this particular study on FV intake, children with parents who were highly involved in the intervention program responded better to the intervention. This is in line with a previous review on parental involvement. Hingle et al. (91) showed that directly involving parents to dietary interventions showed promising results. Despite this finding, the current review provides insufficient evidence on the moderating effects of parental involvement to draw strong conclusions.

In contrast to general expectations, baseline body weight status (e.g., obese vs. normal), ethnicity, SES and age did not yield remarkable, consistent moderating effects. Considering the fact that the lower SES and ethnic minority children have higher risk for obesity and undesired EBRB habits, more high quality research is especially needed to look at the moderating effect of SES and ethnicity. Undoubtedly, the preferred public health option is not having a moderating effect, but a good quality intervention that is highly effective across different subgroups. Due to the lack of information concerning the power of the applied moderation analyses we cannot state with confidence that lack of significant moderators in our review indeed means lack of existing moderating effects.

The psychosocial variables (i.e., intention, attitude and perceived behavior control) moderated the intervention effect on PA level in one study in which children benefitted more who had lower baseline values on these variables. Since these moderators were only analyzed in one study, the generalizability of these results is limited.

### Methodological issues

The overall quality of the conducted moderation analysis of the studies included in this review was unsatisfactory and the methodology needs more careful considerations in future studies. First, many studies conducted stratified analysis of the intervention effect within each subgroup without an appropriate interaction test *(n =* 27). This major shortcoming was a reason to exclude studies from our review. Testing several subgroups simultaneously increases the probability of finding significant results due to chance alone (13,92). For example, categorizing age into four age groups yields four statistical tests to examine the intervention effect on the same outcome variable (92), increasing the likelihood of finding a Type I error (false positive result). The probability of one or more false positive results is about 5% in one test, and increases to 10% for two tests (e.g., two gender groups), and to 14% for three tests (e.g., three age groups) (92). In contrast, using a single interaction term produces a single test and should therefore be favored above subgroup analyses (92).

Second, the fact that many studies did not find a moderating effect, could be explained by a lack of important moderators or a too low power in the studies in order to be able to detect a moderating effect. Moderator analyses often have poor statistical power. Factors that determine the power of a study to detect interactions are the sample size, the size of the moderating effect, the equality of subgroup sizes, measurement error in the variables that constitute the interaction term and categorization of a truly continuous variable (11,93). Categorization of continuous variables leads also to loss of effect sizes and statistical power, and it would likely result in the loss of information about individual differences within the groups (94). Conducting a power analysis before the start of the study helps researchers to maximize statistical power by design and measurement choices. Aguinis (11) provided the statistical software programs for power calculation for categorical moderators. Jaccard et al. (14) provided tables for estimating power for interactions. The most certain strategy to increase power is recruiting the largest sample available (95). This can cause a very large sample size and can be impractical. Another alternative is choosing measures with high reliability since measurement error of variables negatively influences the estimated effect sizes for the interaction term and consequently the power (9,11). Another strategy is to increase Type I error (α) rates by increasing their p-value from the typical 0.05 to for example 0.10 when conducting a moderation analysis (95). This is also preferable due to the fact that moderating effects are often small. Looking at *p*-values, however, is risky, since accepting the null hypothesis when statistical significance is not found has created difficulty in bringing out interaction effects (96).

The third methodological issue that needs improvement is related to the homogeneity of error variance that has an effect on *p*-values of the interaction tests. Homogeneity of error variance is reflected in the distribution of the residuals of the regression analyses. Among the included studies only one study (66) checked the homogeneity of error variance. Researchers should be aware of the fact that when they violate the assumption of homogeneity, the chance of finding a Type I (false positive result) or Type II (false negative result) error increases, depending on the specific sample. Consequently, the results of moderation analyses cannot be trusted (11,18). Homogeneity can be checked with a visual examination of the distributions of residuals along the regression line in a simple scatter plot separately for moderator groups, by a statistical web-based program provided by Aguinis (11) or by a Levene test in ANOVA. When the error variances are not homogenous, the weighted least square approach can be applied as an alternative to ordinary least square regression. This approach corrects the heterogeneity of error variances by obtaining a single weight for each group (97). In addition, applying structural equation modelling (SEM) is a good alternative since SEM analyses do not require the assumption of homogeneity (11,95).

Fourth, potential moderators should be based on a theory, prior findings or literature reviews. In the current literature review we found that only a very small number of studies fulfilled this requirement. When researchers conduct moderation analyses without a rationale, they may be tempted to analyze all variables available as potential moderators, this will increase the chance of finding false positive results (98).

### Limitations and strengths

Our findings should be interpreted in light of methodological strengths and weaknesses. The strength of our review was the extensiveness of literature search as well as being the first paper that assessed the quality of moderation analysis in school based-interventions aimed at EBRBs among youth. We also evaluated the methodological quality of included studies, which was not done by the previous review by Kremer et al. (15). Although our review covered a wide range of school-based obesity prevention interventions, most studies were limited mainly in terms of their methodological quality.

## Recommendations for future studies

### Methodological implications

By including a large enough sample size, determined *a priori* by a power calculation, including balanced subgroups, and using reliable measures with continuous scales (rather than artificially dichotomized scales) researchers may increase the chance of correctly estimating a moderating effect. Reporting moderation analysis in the abstract would increase knowledge building and enable easy access to the study by other researchers aimed at exploring moderators (98).

Researchers who want to explore moderation effects should restrict their investigations to a specific rationale and avoid analyzing variables without a rationale. One other important issue is reporting/ publication bias. Researchers may have applied many tests but report only the significant ones. Another line of research is applying SEM in order to explore moderation effects in studies including latent variables measured by multiple indicators. This statistical method has, when compared to the more traditional regression analyses, the advantage on power that it corrects for measurement error (95,99). An example is reported by Hopwood (100).

### Theoretical implications

The potential moderators stimulate a search for theories and underlying mechanisms why an intervention has differential effects. Moderating effects could also serve to test an underlying theoretical model. Current behavioral change theories do not consider moderating effects that need to be taken into account in the development or improvement of theories. Regarding the importance of contribution of psychosocial factors in health behaviors, information is necessary concerning moderation effects of psychosocial variables. A greater understanding of why different subgroups respond in different ways to an intervention should be further explored. Different subgroups may benefit from different intervention strategies. Therefore, future investigations for varying types of intervention strategies need to be conducted in both qualitative and quantitative studies. Potential moderation effects, such as gender differences in intervention effects, suggest the need for separate programs or at least special considerations for boys and girls that can be provided by tailored intervention messages.

## Conclusion

This systematic review leads to two conclusions. First, the systematic review cannot be viewed as conclusive due to the inconsistent results found in the included studies and the small numbers of significant moderation effects. However, gender (female) and unfavorable baseline values are the most prominent moderators of the intervention effects. The further investigation of underlying mechanisms will help inform the delivery of interventions to those who might benefit the most. Second, the overall methodological quality of the included studies was moderate. The quality of the moderation analysis needs improvement in future studies.
