# Supplementary material for: For whom and under what circumstances do school-based energy balance behavior interventions work? Systematic review on moderators
Source: Int J Pediatr Obes. 2011 Jun 9;6(2_2):e46–57. doi: 10.3109/17477166.2011.566440 (PMC3190836; doi:10.3109/17477166.2011.566440)
Supplement: Supplementary Tables. [file SD1.pdf]

Online Table 1. Online search strategy for the database of Pubmed

**# 1 Children**
"child"[MeSH] OR child* OR schoolchild* OR "adolescent"[MeSH] OR adolescen* OR boy OR boys OR girl OR girls OR youth OR youths OR teen OR teens OR teenager* OR puberty OR pupils
**# 2 Energy Balance-Related Behaviours**
"physical activity" OR exercise OR exercising OR sport OR active OR inactive OR activities OR activity OR walking OR cycling OR sedentary OR computer OR television OR TV OR internet OR games OR gaming OR recreation OR play OR viewing OR nutrition OR food OR diet OR intake OR beverages OR snack OR "soft drink" OR softdrink OR fruit OR vegetable OR drink OR soda OR breakfast OR supper OR lunch OR meal OR dinner OR tea OR fat OR "5-a-day" OR eating OR sitting OR obese OR obesit* OR overweight OR "body mass" OR "body weight" OR "weight gain" OR "weight loss" OR BMI
**# 3 School-Based Intervention**
(prevention OR intervention OR promotion OR trial OR program*) AND (school OR school* OR "school-based" OR education OR grade)
**# 4 Filter of unrelated studies**
NOT infant NOT neonatal NOT alcohol NOT cigarette NOT tobacco NOT cannabis NOT marijuana NOT drug NOT cocaine NOT HIV NOT AIDS NOT condom NOT sexual NOT anorexia NOT bulimia NOT binge NOT autism NOT autistic NOT steroid NOT menopause NOT osteopenia NOT osteoporosis NOT menstruation NOT HRT NOT Schizophrenia NOT mental NOT depression NOT dementia NOT injury NOT HPV NOT vaccine NOT allergy NOT folate NOT folic NOT dental NOT caries NOT oral NOT teeth NOT sleep NOT apnea NOT asthma NOT tumour NOT cancer NOT chemotherapy NOT lymphoma NOT carcinoma NOT hepatitis NOT sun NOT dermatitis NOT eczema NOT acne NOT celiac NOT bullying NOT suicide NOT migraine NOT headache NOT kidney NOT infection NOT hyperactivity NOT cerebral NOT sickle NOT helicobacter NOT arthritis NOT pregnancy NOT contraceptive NOT fertility NOT dysmenorrhea NOT dysmenorrhoea NOT spina NOT fatigue NOT spondylitis NOT thalassemia NOT alzheimer NOT phenylketonuria NOT influenza NOT anaemia NOT iron NOT deficiency NOT malaria NOT measles NOT polio NOT traffic NOT helmet NOT immunization NOT vaccination NOT surgery NOT hygiene NOT gambling NOT fracture NOT stress NOT crohn
**End Search # 1 AND # 2 AND # 3 AND #4**

Online Table 2. Quality assessment of the school-based interventions aimed at energy balance-related behaviors that conducted moderation analysis.

	**Quality criteria**<br><br>**Study**	**Year**	Randomization performed	Groups similar at baseline (regarding age, gender, BMI, outcome measures)	Eligibility criteria specified	Point estimates and variability measures of outcome	Intention-to-treat analyses included	Drop-out rate non selective	Theoretical framework cited	Did reliability and validity checked for outcome measure?	Was a rationale (evidence or theory) for the interaction provided?	**Was power calculated and was it adequate to detect moderation?**	Was the assumption of homogeneous (error) variances across groups checked?	**Total score**
1	Baranowski[20]	2000	+	+	+	+	+	+	+	+	-	-	-	8
2	Haerens[41]	2007	+	?	+	+	+	+	+	+	+	-	-	8
3	Young[79]	2006	+	+	+	+	+	?	+	+	+	-	-	8
4	Bere[24]	2006	+	+	+	+	-	+	+	+	-	-	?	7
5	Bere[23]	2006	+	+	+	+	-	+	+	+	-	-	?	7
6	Hill[44]	2007	+	+	+	+	+	+	+	-	-	-	-	7
7	Simon[66]	2008	+	-	+	+	+	-	+	+	-	-	+	7
8	Perry[60]	1998	+	+	+	+	-	+	+	+	-	-	-	7
9	Verstraete[74]	2007	+	+	+	+	-	+	-	+	+	-	-	7
10	Dzewaltowski[30]	2009	+	+	+	+	-	-	+	+	-	-	-	6
11	Goran[36]	2005	+	-	+	+	?	+	+	+	-	-	-	6
12	Haerens[38]	2007	+	+	+	+	-	?	+	+	-	-	-	6
13	Haerens[39]	2007	+	+	+	+	-	?	+	+	-	-	-	6
14	Himes[45]	2003	+	+	+	+	-	?	+	+	-	-	-	6
15	Kipping[49]	2008	+	+	-	+	+	?	-	+	+	-	-	6
16	Manios[54]	1999	+	?	+	+	-	+	+	+	-	-	?	6
17	Manios[53]	1999	+	?	+	+	-	+	+	+	-	-	?	6
18	Martens[56]	2007	+	?	+	+	+	-	+	+	-	-	-	6
19	Te Velde[72]	2008	+	-	+	+	+	-	+	+	-	-	-	6
20	Verstraete[76]	2007	+	+	+	+	-	+	-	+	-	-	-	6
21	Verstraete[75]	2006	+	+	+	+	-	+	-	+	-	-	-	6
22	Baranowski[21]	2003	+	-	+	+	-	-	+	+	-	-	-	5
23	Burke[26]	1996	+	+	+	-	-	+	-	-	+	-	-	5
24	Cardon[28]	2009	+	-	+	+	-	+	-	+	-	-	-	5
25	Cullen[29]	2005	+	-	+	+	?	-	+	+	-	-	-	5
26	Foster[32]	2008	+	-	+	-	+	+	-	+	-	-	-	5
27	Gentile[34]	2009	+	-	+	+	+	?	+	-	-	-	-	5

28	Going[35]	2003	+	-	+	+	-	?	+	+	-	-	-	5
29	Haerens[40]	2007	+	?	+	+	-	?	+	+	-	-	-	5
30	Harrison[42]	2006	-	+	+	+	?	?	+	+	-	-	-	5
31	Hendy[43]	2007	+	+	+	-	-	?	-	+	+	-	-	5
32	Luepker[52]	1996	+	+	+	+	?	-	+	-	-	-	-	5
33	McKenzie[57]	2001	+	?	+	+	?	?	+	+	-	-	-	5
34	Moore[58]	2008	+	+	+	+	-	?	-	+	-	-	-	5
35	Perry[61]	2004	+	+	-	-	-	+	+	+	-	-	-	5
36	Robinson[63]	1999	+	-	-	+	+	?	+	+	-	-	-	5
37	Shilts[65]	2009	+	-	+	-	-	+	+	+	-	-	-	5
38	Singh[67]	2009	+	-	+	+	-	-	+	+	-	-	-	5
39	Stevens[68]	2003	+	?	+	-	+	?	+	+	-	-	-	5
40	Perry[59]	1998	+	+	-	-	-	+	+	+	-	-	-	5
41	Salmon[64]	2008	+	-	+	-	+	?	+	-	+	-	-	5
42	Webber[77]	2008	+	+	+	-	+	?	+	-	-	-	-	5
43	Ashfield-Watt[19]	2008	+	-	+	+	-	-	-	+	-	-	-	4
44	Barnett[22]	2009	+	?	+	-	-	-	+	+	-	-	-	4
45	Bere[25]	2005	+	-	+	+	-	-	-	+	-	-	?	4
46	Ernst[31]	1999	+	+	-	+	-	?	-	+	-	-	-	4
47	Graham[37]	2008	-	+	+	+	-	-	-	-	+	-	-	4
48	Kelder[48]	1995	-	+	+	-	-	?	+	+	-	-	-	4
49	Loucaides[50]	2009	+	-	+	+	-	?	-	+	-	-	-	4
50	Marcus[55]	2009	+	-	+	-	+	+	-	-	-	-	-	4
51	Reinaerts[62]	2007	+	-	+	+	-	-	+	?	-	-	-	4
52	Wechsler[78]	1998	+	+	+	-	-	?	-	+	-	-	-	4
53	Butcher[27]	2007	+	?	+	-	-	?	-	+	-	-	-	3
54	Horne[46]	2004	-	?	+	+	-	?	-	+	-	-	-	3
55	Horne[47]	2009	+	+	?	-	-	?	-	+	-	-	-	3
56	Stock[69]	2007	-	-	+	+	-	?	-	+	-	-	-	3
57	Tak[70]	2007	-	-	+	+	-	-	-	+	-	-	-	3
58	Tak[71]	2009	-	-	+	+	-	-	-	+	-	-	-	3
59	Vandongen[73]	1995	+	-	+	+	-	-	-	-	-	-	-	3
60	Frenn[33]	2003	-	-	-	-	-	?	+	+	-	-	-	2
61	Lowe[51]	2004	-	-	+	-	-	?	-	+	-	-	-	2
	**TOTAL**		52	27	54	44	15	19	37	51	8	0	1	Mean=5.1

Yes= +, No= - , Don’t know=?

Online Table 3. Characteristics of the included school-based interventions aimed at energy balance-related behaviors.

	Study (Year) (Quality Score)	Sample	Design	Assessment	Theory	EBRBs Outcome (reliability and/or validity)	Intervention effect on outcome	Moderator tested	Results of moderator test	In case of moderators: results of stratified analyses
	**DIETARY INTERVENTIONS**									
1.	Ashfield-Watt[19] (2008) (low:4)	New Zealand 20 primary schools n=2032 %female=50.1 Mean age=8.8 Drop-out=44.0%	RCT I: 10 weeks, free fruit (at least one piece per school day) C: No intervention	Baseline; 10 and 16 weeks post-baseline	-	Fruit intake per school day by Day in the Life Questionnaire (DILQ) (kappa=0.85-0.92)	Significant intervention effect on fruit intake at 10 **weeks**	- Gender - Age - Ethnicity	NS NS NS	
2.	Baranowski[20] (2000) (medium:8)	USA (Gimme 5) 16 elementary schools n=1732 %female=NR Mean age=NR Drop-out=32.3%	RCT I: 6 weeks/year (for 2 years), curriculum, parent involvement, videotapes and daily newsletters to home, point-of-purchase education C: No intervention	Baseline; 12 and 24 months post-baseline	SCT	FJV consumption by 7 day food record (daily servings) (r=0.80-0.98, ICC for F=0.72-0.76, ICC for V=0.59-0.69)	Significant intervention effect on combined FV intake and vegetables alone at the 1st year	- Gender - Ethnicity	NS NS	
3.	Baranowski[21] (2003) (medium:5)	USA (Squire's Quest!) 26 elementary schools n=1578 %female=50.9 Mean age= 8.3 Drop-out=5.3%	RCT I: 5 weeks, Interactive multimedia game, goal setting C: No intervention	Baseline; 5 weeks post-baseline	SCT	FJV consumption by 4 nonconsecutive days food intake recording software system (r=0.44-0.59)	Significant intervention effect on fruit intake, regular vegetables intake and total FJV intake	- Gender - Age - Ethnicity	NS NS NS	
4.	Bere[24] (2006) (medium:7)	Norway (Fruit and Vegetables Make the Marks-FVMM) 19 schools (9	RCT I: 7 months, curriculum, parental involvement, paid	Baseline; 8 and 20 months post-baseline	SCT	FV consumption by 24 hour recall (test-retest r=0.62-0.83, validity r=0.21-	NS	- Gender	NS	

		intervention)<br>n=450<br>%female=54.2<br>Mean age=11.3<br>Dropout=31.0%	school fruit programme (only at 4 schools)<br>C: 3 schools in paid school fruit programme, others no intervention			0.32)				
5.	Bere[23]<br>(2006)<br>(medium:7)	Norway (Norwegian School Fruit Programme + FVMM)<br>19 schools (9 intervention)<br>n=577,<br>%female=47.6<br>Mean age=11.3<br>Drop-out=10.4%	RCT<br>I: 7 months curriculum, parental involvement, school fruit programme (1st year free, 2nd year paid fruit only in 4 schools)<br>C: 1st year 2, 2nd year 3 schools in school fruit programme, others no intervention	Baseline; 8 months and 20 months post-baseline	SCT	FV consumption by 24 hour recall and food frequency questionnaire (test re-test r=0.62-0.83, validity r=0.21-0.32)	Significant intervention effect on FV intake in both follow-ups	- Gender<br>- Habitual FV intake<br>- Preferences of FV<br>- SES	NS in Year 1, Significant in Year 2<br>NS<br>NS<br>NS	Stratified by gender;<br>Boys > Girls (NS)
6.	Bere[25]<br>(2005)<br>(low:4)	Norway (Norwegian School Fruit Programme)<br>38 schools (9 free fruit, 9 paid fruit, 20 control)<br>n= 922,<br>%female=50.1<br>Mean age=12.3<br>Drop-out=13.8%	3-arm RCT<br>I:1- free fruit programme,<br>2- paid fruit programme<br>C: No intervention	Baseline; 8 months post-baseline	-	FV consumption by 24 hour recall and food frequency questionnaire (test re-test r=0.62-0.83, validity r=0.21-0.32)	Significant intervention effect among free fruit group compared to paid and no fruit group<br>Significant intervention effect among subscribers at the paid fruit group compared to non-subscribers	- Gender<br>- Habitual FV intake<br>- SES	NS<br>Significant in FV intake at school and snacks<br>Significant in soda/candy/chips intake	Stratified by habitual FV intake;<br>Low habit > high habit<br>Stratified by SES:<br>Low SES > high SES
7.	Cullen[29]<br>(2005)<br>(medium:5)	USA (Squire's Quest!)<br>26 elementary schools (13 intervention)<br>n=1578,<br>%female=50.9<br>Mean age= 8.3<br>Drop-out=5.3%	RCT<br>I: 5 weeks Interactive multimedia game, goal setting<br>C: No intervention	Baseline; 5 weeks post-baseline	SCT	FJV consumption at specific meals and snacks by 4 days food intake recording software system (r=0.44-0.59)	Significant intervention effect on servings of fruit and 100% fruit juice at snacks, and regular vegetables at lunch	- Gender<br>- Age<br>- Ethnicity<br>- SES	NS<br>NS<br>NS<br>NS	
8.	Hendy[43]	USA (Kids	3-arm RCT	Baseline;	-	FV consumption	Significant	-Weight	Study phase x	

	(2007) (medium=8)	choice programme) 1 school n=313, %female=46.2 Mean age=8.0 Drop-out=12.1%	I:1) Changes in school lunch procedures, token reinforcement for vegetables, 2) Changes in school lunch procedures, token reinforcement for fruits C: Changes in school lunch procedures	2 weeks and 7 months post-baseline		by lunch observations, 3 days a week (inter-observer r= 0.92 for fruit consumption and r=0.94 for vegetable consumption)	intervention effect on FV intake at both follow-ups in both reinforcement conditions	status -Food type provided	Food type provided x Weight; NS	
9.	Horne[46] (2004) (low=3)	UK (The Food Dudes) 2 primary schools n=749, %female=NR Age range=5-11 Drop-out=NR	Quasi experimental I;16 days, peer modelling videos, rewards, home packs, letters, free FV availability at school lunch for 24 days, also at snacktime for 5-7 y olds, 4 months maintenance without videos C:No Food Dudes program but free availability of FV at lunch and snacktime	Baseline; 1 month and 5 months post-baseline	-	FV consumption by lunchtime observation (kappa coefficient=0.89-0.96) snacktime consumption by weighed FV before and after consumption (r=NR) and home consumption by parental recall (r=NR)	Significant intervention effect on lunchtime, snacktime and home FV consumption at both follow-ups	-Free food type (free fruit, free vegetable) - Age (5-7y, 7-11y)	Significant on lunchtime FV consumption<br><br>School x Study phase x Food x Age; Significant on lunchtime FV consumption	Stratified by food type and age group; Fruit and Vegetable both significant Younger > Older
10.	Horne[47] (2009) (low=3)	Ireland (The Food Dudes) 2 primary schools n=435, %female=NR, Age range=4-11 Drop-out=NR	RCT I;16 days, peer modelling videos, rewards, homepack, letters, free FV availability at school lunch, maintenance phase without videos, supporting parents for providing FV in lunchboxes, rewards C:No intervention	Baseline; 12 months post-baseline	-	Consumption of FV at lunchboxes by Lunchbox Measures 5 days (r=NR) Consumption of free FV provided by school by School-provided foods measures 4 days (r=NR)	Significant on both consumption of lunchboxes FJV and school provided FV	- Food type (fruit, vegetable)<br><br>- Age (4-7y, 7-11y)	Time x Group x Food ; Significant for total consumption of school-provided FV NS	Stratified by food; Fruit and Vegetable both significant

11. Lowe[51] (2004) (low=2)	UK (The Food Dudes) 3 primary schools n=402, %female=NR, Age range=4-11 Drop-out=NR	Quasi experimental I;16 days, 1) peer modelling videos, rewards, letters, free FV availability at school lunch at snacktime, 2) 16 days, peer modelling videos, rewards, letters, free FV availability at school lunch at snacktime, homepacks C:No intervention	Baseline; post interventi on (Time interval was not reported)	-	FV consumption by lunchtime observation (kappa coefficient=0.96-0.99) snacktime consumption by weighed measure (r=NR) and home consumption by parental recall (r=NR)	Significant intervention effects on lunchtime, snacktime consumption and weekdays consumption outside school.	- Age (4-7y, 7-11y)	Significant on lunchtime fruit consumption	<u>Stratified by age;</u> Younger > Older
12. Martens[56] (2007) (medium=6)	The Netherlands (Krachtvoer) 18 schools n=1613 %female=NR Age range=12-14 Drop-out=22.5%	RCT I:curriculum, materials (lunch-boxes, take home bag, magazine, posters, postcards, video, website, newsletters, taste-testing) C: No intervention	Baseline; 3 months post-baseline	ASE SM	FJ consumption by a self-administered 24-hour recall (r=0.53-0.64) Snack consumption by a short food frequency questionnaire, the Fat list (r=0.60) Breakfast consumption by 1 item	Significant effect on fruit consumption	- Gender<br>- Age<br>- Baseline value (centered-low, medium, high)	Significant on fruit frequency Significant on total fat consumption at breakfast Significant on all outcome variables	<u>Stratified by gender and age</u>; NR <u>Stratified by baseline value</u>; - Fruit consumption Low, medium > high - Fruit juice consumption; High > Low -Snack consumption; High > Low -Breakfast saturated fat intake; High > Low
13. Moore[58] (2008) (medium=5)	UK 43 primary schools n=1632 %female=51.3 Age range=9-11 Drop-out=NR	RCT I:1 school year, fruit tuck shops at schools C: No intervention	Baseline; 9 months post-baseline	-	Fruit, sweet (sweets, chocolate, biscuits) and savory snacks (crisps) consumption at school and 24 h by a single-day	Significant effect on fruit intake as snacks at school	-School food policy	Significant on fruit consumed at school	<u>Stratified by school policy</u>; Fruit only policy > No policy

					computerized 24-hour recall questionnaire (kappa=0.29)				
14. Perry[61] (2004) (medium=5)	USA (5-a-day Cafeteria Power Plus) 26 schools n=1668 % female=49 Mean age=NR Drop-out=30%	RCT I: 2 school year, school food service change, social support C: No intervention	Baseline; 2 years post-baseline	SCT HBPM	FV consumption at lunch by lunch observation (r=NR)	Significant intervention effect on total FV intake	- Gender<br>- Age	NS<br>NS	
15. Perry[59] (1998) (medium=5)	USA (5-a-day Power Plus) 20 elementary schools n=536 % female=50.0 Mean age=10.0 Drop-out=17.7%	RCT I: Behavioural curricula, parental involvement, school food service changes, industry support C: No intervention	Baseline; 12 months post-baseline	SLT	FV consumption and total dietary intake (total energy, fat, saturated fat) by 24-h recall (r=0.45-0.79) and lunch observation (r=NR)	Significant intervention effect on FV consumption	- Gender<br>- Ethnicity	Significant on vegetable consumption<br>Significant on total fat and saturated fat intake	Stratified by gender; Girls > Boys<br>Stratified by ethnicity; Asians, African-Americans > Hispanics, Caucasian
16. Reinaerts[62] (2007) (low=4)	The Netherlands 12 primary schools n=1730 % female=51.5 Mean age=9.0 Drop-out=45.7%	3-arm RCT I: 9 months I1) Free FV distribution (twice a week), I2) Classroom curriculum, parental involvement C: No intervention	Baseline; 9 months post-baseline	SCT ELM HBM HT	FV consumption by pre-structured 24-h food recall, FFQ - parents reported (r=NR)	Both intervention arms had significant effect on FV consumption	-Gender<br>-Ethnicity<br>-Age	Significant on vegetable intake<br>Significant on vegetable intake and 24 h FJV intake<br>Significant on vegetable intake and 24 h FJV intake	Stratified by gender; Girls > Boys<br>Stratified by ethnicity; Non-Native > Native<br>Stratified by age; Older > Younger
17. Tak[70] (2007) (low=3)	The Netherlands (The Dutch SchoolGruiten Project) 55 schools n= 1328 % female=55 Mean age=10.0 Drop-out= 28.2%	Quasi experimental I: 2 years, Free piece of fruit or vegetable (twice a week), school curriculum to increase knowledge and skill related to F&V consumption (not obligatory) C: No intervention	Baseline; 1 year post-baseline	-	FV intake by FFQ both from children (r=0.47-0.84) and parents (validity r=0.40-0.53, test-retest r=0.47-0.84)	Significant intervention effect on vegetable intake of non-Western children Significant intervention effect on fruit intake of Dutch children	-Gender<br>-SES (low, medium, high)	Significant on vegetable intake of Dutch children<br>Significant on fruit intake of Non-western children	Stratified by gender; Girls > Boys<br>Stratified by SES; NS difference

18. Tak[71] (2009) (low=3)	The Netherlands (The Dutch SchoolGruiten Project) 55 schools n=1328 % female=55.1 Mean age=10.0 Drop-out: 41.9%	Quasi experimental I: 2 years, Free piece of fruit or vegetable (twice a week), school curriculum to increase knowledge and skill related to F&V consumption (not obligatory) C: No intervention	Baseline; 1 year and 2 years post-baseline	-	FV intake by FFQ both from children (r=0.47-0.84) and parents (validity r=0.40-0.53, test-retest r=0.47-0.84)	Significant increase in fruit intake at the 2nd follow up both in children and parent reports No significant effect on vegetable intake	-Gender<br>-Ethnicity<br>-SES<br>-Region of residence	NS<br>NS<br>Significant on child-reported fruit intake<br>NS	Stratified by SES;NS difference
19. Te Velde[72] (2008) (medium=6)	Norway, The Netgerlands, Spain (Prochildren) 62 schools n=1801 %female=61.2 Age range=10-13 Drop-out=23.8%	RCT I: 2 school years, classroom component (activity worksheets and computer-tailored feedback), school component (free or paid FV), family component (homework assignments, newsletters, parent version of computer-tailored tool), optional component (community participation) C: No intervention	Baseline; 20 and 32 months post-baseline	SCT<br>ASE<br>TTI<br>SEF	Total and separate FV intake by 24 h recall, and by FFQ (validity r=0.40-0.53, test-retest r=0.47-0.84)	Significant effect on total and separate FV intake at the 1st follow-up	-Gender<br>-Country	NS<br>Significant at 2nd follow-up at total FV intake and total fruit intake	Stratified by country Norway > Netherlands, Spain
20. Wechsler[78] (1998) (low=4)	USA 6 elementary schools n=6902 %female=NR Mean age=NR Drop-out= None (Unit of analysis was school)	RCT I:7-10 days, social marketing techniques (e.g. taste tests, promotion incentives, product positioning), auditorium session, flyers C: No intervention	Baseline; 7 to 8 days, 3 to 4 months post-baseline	-	Lunchtime low-fat milk consumption by sampling discarded milk cartons (inter-rater r=0.99)	NS on milk consumption	-Gender<br>-Age (younger (1st-2nd grades) vs. older (3rd-4th grade))	NS<br>NS on consumption	

**PA INTERVENTIONS**									
21. Barnett[22] (2009) (low:4)	Australia (The Physical Activity and Skills Study) 18 schools n=928 %female=53.0 Mean age=10.1 Drop-out=70.3%	Quasi-experimental I: 1 year, Modification of existing PE classes C:No intervention	Baseline; 1 year and 6 years post-baseline	-	PA in a week by the Adolescent Activity Recall Questionnaire (APARQ) (r=0.35-0.88, kappa=0.25-0.74)	NS	- Gender<br>- Age	NS<br>NS	
22. Butcher[27] (2007) (low=3)	UK 3 schools (1 per condition) n=177, %female= 45.8 Age range= 7-11 Drop-out=20.3%	3-arm RCT I:1- Feedback on pedometer steps 2- Feedback and information on pedometer steps C: No intervention	Baseline; 1 week post-baseline	-	Daily step counts by pedometer (ICC=0.90-0.94)	Significant intervention effect on daily step counts in feedback + information group compared to other groups.	- Gender	NS	
23. Cardon[28] (2009) (low=4)	Belgium 40 pre-schools (10 per condition) n=636, %female=47.8 Mean age=5.3 Drop-out= 8.3%	RCT I:1-play equipment provided, 2-painted markings on the playground, 3-play equipment + painted markings on the playground C:No intervention	Baseline; 3 months post-baseline	-	PA level during recess by accelerometers	NS	- Gender<br>- Age<br>- Pre-test values<br>- Average recess time	NS<br>NS<br>NS<br>NS	
24. Ernst[31] (1999) (low=4)	USA (Promoting Lifetime Activity for Youth-PLAY) 5 elementary schools n=NR %female=NR Mean age=NR Drop-out=NR	RCT I:12 weeks, 15 min PA break each school day with classroom teachers support, goal setting and recording handbooks C: 15 min activity break without teachers support, recording handbooks	Baseline; 4 weeks and 12 weeks post baseline	-	PA level by 7 days PA Questionnaire for Older Children (PAQ-C) (test-retest r=0.75-0.82) (concurrent validity r=0.45-0.53)	Significant intervention effect on PA level in intervention group	-Baseline activity level	NS	
25. Goran[36] (2005)	USA (IMPACT) 4 elementary	RCT I: 8 weeks,	Baseline; 2 and 4	SCT	PA by 5 days uniaxis	NS on total PA Significant effects	- Gender	Significant on LPA	<u>Stratified by gender;</u>

	(medium=6)	schools<br>n=209,<br>%female= 51.0<br>Mean age=9.5<br>Drop-out=40.7%	Interactive educational learning game<br>C:Popular educational CDs (unrelated to health)	months post-baseline		accelerometer (r=0.80-0.92)	on MVPA and LPA			Girls > Boys
26.	Graham[37] (2008)<br>(low=4)	USA<br>2 high schools<br>n=122<br>%female=100<br>Mean age=15.0<br>Drop-out:2.5%	Quasi experimental<br>I: 1 school year intervention: 4 weekly supervised exercise sessions + 1 weekly health education C: no intervention	Baseline;<br>9 months post-baseline	-	PA- from 3 day PA recall aggregated into moderate (MPA) and vigorous (VPA) activities (r=NR)	Significant intervention effects on VPA	-TV viewing (high vs. low)	Significant on VPA	Stratified by TV viewing;<br>High > Low
27.	Harrison[42] (2006)<br>(medium=5)	Ireland (Switch off-Get Active)<br>9 primary schools n= 312,<br>%female=43.3<br>Mean age=10.2<br>Drop-out=9%	Quasi experimental<br>I: 16 weeks, health education, diaries signed by parents, homeworks<br>C: No intervention	Baseline;<br>16 weeks post-baseline	SCT	PA and screen time by 1-day Previous day PA recall (test-retest r=0.98, validity against accelerometer r=0.88)	Significant intervention effect on physical activity but not on screen time	-Gender<br>- BMI	NS<br>NS	
28.	Hill[44] (2007)<br>(medium=7)	UK<br>1 secondary school<br>n=620<br>51% girls<br>Mean age: 16.9<br>Drop-out: 16%	4-arm RCT<br>I: 1) Leaflet; 2) Leaflet + motivational incentive and quiz; 3) Leaflet + implementation intention prompt<br>C: No intervention	Baseline;<br>3 weeks post-baseline	TPB<br>ELM	Exercise- from 1 item weekly exercise measure (r=NR)	Significant intervention effects on weekly exercise.<br>No differences between interventions	- Intention<br>- PBC<br>- Attitude<br>- Normative beliefs	Significant<br>Significant<br>Significant<br>NS	Stratified analysis;<br>Lower > Higher
29.	Loucaides[50] (2009)<br>(low=4)	Cyprus<br>3 schools<br>n= 247<br>%female=NR<br>Mean age=11.1<br>Drop-out=7.7% (break time), 6.1% (after school)	RCT<br>I:1) play space for team games, playground markings, providing equipment 2) play space for team games, providing equipment<br>C: No intervention	Baseline;<br>4 weeks post-baseline	-	PA during school breaks and after school period by pedometer (r=0.53-0.81)	Significant intervention effect on break-time activity in both intervention groups compared to control school	- Gender	NS	

30. Salmon[64] (2008) (medium=5)	Australia (Switch-Play) 3 schools n=295 %female=51.0 Mean age=10.7 Drop-out=12%	4-arm RCT I: 9 months, I1) behavioural modification (BM) group; classroom based, parent involvement, I2) Fundamental movement skills (FMS) group; funny games for 6 movement skills, I3) BM+FMS C: No intervention	Baseline; **9** and 23 months post-baseline	SCT BCT	Physical activity by accelerometers (r=NR) Self-reported screen behaviours by a questionnaire (ICC>0.6)	Significant intervention effect on physical activity in BM and FMS intervention arms at both time points Significant intervention effect of BM on TV viewing in undesired direction at both time points	-Gender	Significant on movement counts, MPA and VPA and screen behaviours	<u>Stratified by gender</u> In FMS and BM+FMS groups Boys > Girls
31. Simon[66] (2008) (medium=7)	France (ICAPS) 8 middle schools n=954 %female=50.0 Mean age=11.6 Drop-out=23.3%	RCT I:4 years, educational classes, debates, opportunities for PA at lunchtime and breaks, sporting events, parental support C:No intervention	Baseline; 8, 20 and 32 months post-baseline	SEF	Self-reported leisure PA and TV/video viewing and active commuting by the Modifiable Activity Questionnaire (ICC=0.71-0.83)	Significant intervention effect on leisure time PA and at all time points TV/video viewing only at 20 months follow-up	-Gender<br>-SES<br>-Initial weight<br>-Sports club participation	NS<br>NS<br>NS<br>NS	
32. Verstraete[76] (2007) (medium=6)	Belgium 16 elementary schools n=810 %female=50.7 Mean age=9.7 Drop-out= 5.7%	RCT I:2 school years, Health-related PE programme, classroom-based health education lessons, an extra-curricular PA promotion programme C: No intervention	Baseline; 20 months post-baseline	-	PA level in leisure time by a PA questionnaire (ICC=0.68-0.93, validity r=0.27-0.44) PA level by a 5 day uniaxis accelerometer (validity r = 0.50-0.74)	Significant effect on MPA and MVPA, and on MPA in leisure time	-Gender	NS	
33. Verstraete[75] (2006) (medium=6)	Belgium 7 elementary schools n=249 %female=48.5 Mean age=10.8 Drop-out= 5.6%	RCT I:2 school years, Health-related PE programme, classroom-based health education lessons, an extra-curricular PA	Baseline; 3 months post-baseline	-	PA level by uniaxis accelerometers at 1 day morning recess and lunch break (validity r = 0.50-0.74)	Significant effect on MPA and VPA at lunch break, on MPA at morning break	-Gender<br><br>-Baseline MVPA	Significant on LPA, MPA and MVPA<br>NS	<u>Stratified by gender;</u> Girls > Boys

		promotion programme C: No intervention						
34.	Verstraete [74] (2007) (medium=7)	Belgium 16 elementary schools n=791 %female=52.2 Mean age=9.7 Drop-out=3.4%	RCT I:2 school years, curricula, PE lessons, health-fitness and skill-fitness activities C: No intervention	Baseline; 20 months post-baseline	-	PA by accelerometer by uniaxis accelerometers (r=NR) PA in PE lessons by SOFIT (intrarater r=0.80-0.99, ICC= >0.95)	Significant effect on PA in PE lessons	-Gender	NS
35.	Webber (2008) (medium=5)	USA (Trial of Activity for Adolescent Girls (TAAG)) 36 middle schools n= 1603(baseline)-3378 (follow-up) %female=100 Mean age=12 Drop-out=5.5% (Analysis unit was school)	RCT I: 2 years, environmental and organizational changes supportive of PA, cues, messages and incentives to be more PA, lessons to enhance behavioural skills C: No intervention	Baseline; 2 and 3 years post-baseline	OLT SCT OCT DIM	PA and sedentary behaviour by 7 days wear of uniaxis accelerometers (r=NR)	Significant intervention effect on MVPA and sedentary behaviour at the second follow-up	-Ethnicity	NS
36.	Young[79] (2006) (medium=7)	USA 1 high school n=221 %female=100 Mean age= 13.8 Drop-out=5%	RCT I: 8 months, curricula, small-group discussion, homeworks, PE curriculum, weekly exercise logs and feedback on it, parental involvement C: Standard PE, newsletters to parents about PA content and general health interests	Baseline; 8 months post-baseline	SAT	PA by self-reported 7 day PA recall (test-retest r=0.59-0.81, validity r=0.76) Sedentary activities (TV viewing, computer, internet using) (test-retest r=0.94)	Significant effect on TV viewing during school days	-Ethnicity	NS

MULTIPLE EBRB INTERVENTIONS

37. Burke[26] (1996) (medium:6)	Australia<br>30 primary schools<br>n=1147<br>%female=43.8<br>Age range=10-12 Drop-out=15.3%	6-arm RCT<br>I:9 months with<br>1- Physical fitness,<br>2-physical fitness+school nutrition,<br>3-school nutrition,<br>4-school + home nutrition,<br>5-home nutrition<br>C:No intervention	Baseline; 10 months post-baseline	-	Nutrient intake (fibre, fat and sugar intake) by 2 days dietary recall (r=NR)	Significant intervention effect on nutrient intake only in RCT arms which included home nutrition.	- Cluster membership (High vs. Low cardiovascular risk)	Significant on sugar intake	<u>Stratified by cluster membership;</u><br>high risk boys > low risk boys<br>in school+ home nutrition group
38. Dzewaltowski[30] (2009) (medium:6)	USA (Healthy Youth Places)<br>16 middle schools n=2211<br>%female=53.0<br>Mean age: 12.4<br>Drop-out: 28%	RCT<br>I: intervention promoting personal and proxy agency to built healthy school environments that promote FV intake and PA<br>C: No intervention	Baseline; 12 and 24 months post-baseline	SCT	PA- from previous day PA recall questionnaire (test-retest r=0.98, validity r=0.88)<br>FV: 24-h Youth/ Adolescent FFQ (r=0.21-0.58)	Significant effect on VPA and MVPA<br>NS on FV intake	- Gender<br>- Ethnicity<br>- SES<br>- BMI	NS on PA<br>NS on PA<br>NS on PA<br>?<br>Results on FV intake not reported	
39. Foster[32] (2008) (medium:5)	USA (School Nutrition Policy Initiative)<br>10 schools<br>n=1349<br>%female=53.7<br>Mean age= 11.2<br>Drop-out=37.4%	RCT<br>I: school self-assessment, nutrition education, nutrition policy, social marketing, parent outreach<br>C: No intervention	Baseline; 24 months post baseline	-	Dietary intake (total energy, total fat, FV intake) by Youth/Adolescent Questionnaire (validity r=0.54, test -retest r=0.21-0.58)<br>PA and sedentary behaviour by Youth/Adolescent Activity Questionnaire (r=NR)	NS on dietary intake and PA<br>Significant intervention effect on total inactivity and television watching	- Gender<br>- Age<br>- Ethnicity	NS<br>NS<br>NS	
40. Frenn[33] (2003) (low=1)	USA<br>2 middle schools n=341<br>%female=NR<br>Age range=12-15 Drop-	Quasi-experimental<br>I: 4 internet and video sessions, a healthy snack and gym labs C:No intervention	Baseline; post baseline (Time interval was not	TM, HPM	Fat intake by Food habits questionnaire (α=0.82)<br>Physical activity by Child	NS	- Gender<br><br><br>- Ethnicity	Gender x Group;<br>Significant on fat intake<br>Gender x Ethnicity x	<u>Stratified by gender and ethnicity</u>;<br>In girls, Asians < other ethnicities

	out=61.9%		reported)		adolescent activity log (α=0.77)		- SES	Group; Significant on fat intake Ethnicity x SES x group; Significant on MVPA	Stratified by ethnicity and SES ; In the low SES group; Native Americans < other ethnicities
41. Gentile[34] (2009) (medium=5)	USA (The Switch) 10 Elementary schools n=1196 %female=53.2 Mean age=9.6 Drop-out= 7.2%	RCT I: Community components as advertising key messages, school and family component as providing materials and goal setting C:No intervention (possible to expose the community component)	Baseline; 7 and 13 months post baseline	SEF	PA by pedometer Screen time (parent and child reported) FV consumption by items from National Youth Risk Behaviour Survey (parent and child reported) (r=NR)	NS on PA level Significant intervention effect on parent reported screen time Significant intervention effect on parent reported FV consumption	- Gender<br>- Family involvement<br>- Weight status	Significant on FV intake and PA level<br>Significant on FV intake<br>Significant on screen time	Stratified by Gender; Girls > Boys Stratified by family involvement: High involvement > low involvement Stratified by weight status; Obese > normal
42. Going[35] (2003) (medium=5)	USA (Pathways) 41 schools n=580 %female= 48.3 Mean age=7.6 Drop-out=52.1%	RCT I:3 year trial with a school curriculum, a PA/PE component, a school food service component, and a family component C:No intervention	Baseline; 3 years post-baseline	SLT	PA by 1 day triaxis accelerometer wearing (r=0.71)	NS	- Site	NS	
43. Haerens[38] (2007) (medium=6)	Belgium 10 secondary schools n=304 %female=70.4 Mean age=13.2 Drop-out=NR	RCT I: interactive computer tailored intervention which aimed dietary fat intake, tailored feedback C:No intervention	Baseline; 13 weeks post-baseline	TM TPB SCT ASE	Dietary fat intake by FFQ (α=0.83, validity r=0.78 against 7-day food diary)	Significant intervention effect on fat intake	- Gender - Type of Education	Condition x Gender x Education; Significant	Stratified analysis by gender and education; In girls; technical-vocational school > general schools
44. Haerens[41] (2007)	Belgium 15 schools	3- arm RCT I: 1 school year	Baseline; 9 months	TPB TM	Total and leisure time PA level by	Significant intervention effect	- Gender	Significant for leisure time	Stratified by gender;

	(medium=8)	n=2840 %female=36.6 Mean age=13.1 Drop-out=14.3%	intervention with 1) individual component: computer tailored PA intervention + environmental component: promoting PA availability, 2) components of intervention arm 1+ parental involvement C: No intervention	post-baseline	SCT	Flemish PA Questionnaire (test-retest r>0.70, validity r=0.43-0.79) PA level by accelerometers (r=0.78-0.80)	on self-reported school-related PA Significant intervention effect on LPA and MVPA measured with accelerometers		active transportation NS for accelerometer data	Girls > Boys
45.	Haerens[39] (2007) (medium=6)	Belgium 10 secondary schools n=281 %female=48.8 Mean age=13.2 Drop-out=?	RCT I: interactive computer tailored intervention which aimed PA, tailored feedback C:No intervention	Baseline; 13 weeks post-baseline	TM TPB SCT ASE	PA level by Flemish PA Questionnaire (test-retest r>0.70, validity against accelerometer r=0.43-0.79)	Significant intervention effect on school related physical activity levels	- Gender - Compliance with the PA guidelines	NS NS	
46.	Haerens[40] (2007) (medium=5)	Belgium 15 schools n=2840 %female=36.6 Mean age=13.1 Drop-out=15.7%	3- arm RCT I: 1 school year interventions with 1) individual component: computer tailored intervention on fat and fruit intake+ environmental component: promoting availability of healthy products 2) components of intervention arm 1 + parental involvement C: no intervention	Baseline; 9 months post-baseline	TPB TM SCT	Dietary fat intake by self-administered questionnaire (r=0.70-0.87, validity r=0.67-0.60 against 7-day food diary) Fruit intake by FFQ (r=0.52-0.82) Soft drink and water consumption by another FFQ (r=NR)	NS	- Gender	Significant on daily fat intake and percentage of energy from fat	<u>Stratified by gender;</u> Girls > Boys in parental support group compared with both other conditions.
47.	Himes[45] (2003) (medium=5)	USA (Pathways) 41 schools n= 470 for lunch	RCT I:3 year trial with a school curriculum, a PA/PE	Baseline; 3 years post-baseline	SLT	Nutrients intake (total energy, fat, protein, carbohydrate,	Significant intervention effects on percentage of energy from total	- Gender	NS	

	observation (n=620 for 24-h dietary recall) %female=48.3 Mean age=7.5 Drop-out=NR	component, a school food service component, and a family component C:No intervention			saturated and polyunsaturated fat, sucrose, fructose, fibre intake) by school lunch observation (r=0.96) and by single 24-hour dietary recall (r=NR)	fat, saturated fat and total carbohydrate by lunch observation and 24-h recall. Significant effects on total energy, protein, total fat, saturated fat and polyunsaturated fat intake by 24-h recall			
48. Kelder[48] (1995) (low=4)	USA (The Class of 1989 Study) n=2376 %female=50.0 Mean age=NR Drop-out=NR	Quasi experimental I: 5 year, peer leaded education program, lunch bags, preparing own newspaper, monitoring aerobic activity, food preparation, community component; mass media and adult education, food labelling C: No intervention	Baseline; 1, 2, 3, 4, 5 and 6 years post-baseline	SCT	PA by self reported hours of exercise per week outside of gym class (test retest r=0.61)	? Results for the total study population were not reported	-Gender	NS except 3rd follow-up	Stratified by gender; Girls > Boys
49. Kipping[49] (2008) (medium=6)	UK (Active for Life Year 5) 19 primary schools n= 604 %female=42.9 Mean age=9.41 Drop-out=23.6% (on activity data), 24.4% (on screen time data)	RCT I:5 months, PA lessons, nutrition lessons, screen viewing lesson C:No intervention	Baseline; 5 months post baseline	-	Time spent doing screen-based activities by a questionnaire (r=0.94) Active transport by 'A Day in the Life' questionnaire (r=NR)	NS	- Gender	NS	

50.	Luepker[52] (1996) (medium=5)	USA (CATCH) 96 elementary schools n=5106 %female= 48.2 Mean age= 8.8 Drop-out=21%	RCT I:3 years, classroom curricula, food service changes, physical education modifications, family participation C: No intervention	Baseline; 1, 2 and 3 years post-baseline	SCT	Total energy, fat, saturated fat, cholesterol intake by a 24 hour dietary recall (r=0.45-0.79) Leisure time PA, TV watching, video gaming by Self-administered physical activity checklist (validity r=0.60-0.76)	Significant effects on percentage of energy intake from fat, protein and carbohydrates, daily saturated fat, polyunsaturated and monounsaturated fat and cholesterol intake and on VPA	- Gender<br>- Ethnicity<br>- Site	NS<br>NS<br>NS
51.	Manios[54] (1999) (medium=5)	Greece 21 primary schools n=579 %female=NR Mean age= NR Drop-out=18.7%	RCT I:3 years, curriculum, PE sessions (2/per week), parental involvement C: No intervention	Baseline; 3 years post-baseline	SLT	3 day physical activity measurement (out of school) by parent reported questionnaire (validity r = .68, test-retest r=0.64) Total energy, fat, protein, carbohydrates, fiber, cholesterol intake by 3 day weighed food record-parent reported (r=NR)	Significant effect on leisure time MVPA	-Gender<br>-Baseline values<br>-Parental education level<br>-Increase in heights<br>-Initial BMI	NS<br>NS<br>NS<br>NS<br>NS
52.	Manios[53] (1999) (medium=5)	Greece 21 primary schools n=579 %female=NR Mean age= NR Drop-out=14.8%	RCT I:3 years, curriculum, PE sessions (2/per week), parental involvement C: No intervention	Baseline; 6 years post-baseline	SLT	3 day physical activity measurement (out of school) by parent reported questionnaire (validity r = .68, test-retest r=0.64) Total energy, fat, protein, carbohydrates intake by 3 day weighed food	Significant effect on leisure time MVPA and on total energy, fat, protein, saturated, monounsaturated and polyunsaturated fatty acids intake.	-Gender<br>- Baseline values<br>-SES<br>-Initial BMI	NS<br>NS<br>NS<br>NS

					record-parent reported (r=NR)				
53.	Marcus[55] (2009) (low=4)	Sweden (STOPP) 10 schools n=1538 (PA), 770 (diet) %female= 50.5 Mean age=8.1 Drop-out=15.9% (PA), 10.1% (diet)	RCT I:4 school years, additional 30 min daily PA, changes at school lunch content, increasing awareness C: No intervention	PA-Once (between 8 months-4 years post-baseline) Diet-Once (4 years post-baseline)	-	Eating habits (FV, fast food, dairy products, nuts, sweets) by a questionnaire, parent-reported (r=NR) PA by 7 day uniaxis accelerometer (r=0.93, validity against direct calorimeter r=0.78-0.80)	Significant effect on high-fat dairy product, sweetened cereals, sweet products	- SES (low vs high)	Significant on parent reported intake of dairy product and fast food	Stratified by SES; Low SES > High SES
54.	McKenzie[57] (2001) (medium=5)	USA (CATCH) n= 96 elementary schools (School is the analysis unit) Drop-out= None	RCT I:2.5 year, physical education modifications (curricular materials, professional development sessions for teachers, feedback and technical support) C: No intervention	Baseline; 6, 12, 18, 24, 30 and 36 months post-baseline	SCT	Physical activity in PE lessons by Systematic observation of PE lessons (r=0.94-0.99)	Significant effect on student's activity levels during PE lessons	-Teacher speciality (PE teacher vs. classroom teacher)<br>-Location (indoor vs. outdoor)	Significant<br>Significant	Stratified by speciality; Classroom teachers > PE teachers<br>Stratified by location; Indoor > Outdoor for VPA, Outdoor> Indoor for walking
55.	Perry[60] (1998) (medium=7)	USA (CATCH) 96 elementary schools n= 1874 %female=50.0 Mean age=8.8 Drop-out=36.7%	RCT I:3 years, classroom curricula, food service changes, physical education modifications C: No intervention	Baseline; 3 years post-baseline	SCT	FV consumption by 24-h recall (r=0.45-0.79)	NS	Gender<br>Ethnicity<br>Site	NS<br>NS<br>NS	
56.	Robinson[63] (1999) (medium=5)	USA 2 elementary schools n=198 %female=46.5 Mean age=8.9 Drop-out=3.0%	RCT I: 6 months, classroom curriculum, newsletters to parents C: No intervention	Baseline; 6 months post-baseline	SCT	Media use by questionnaire (r=0.94), Dietary intake (high-fat food) by 1-day food frequency recall (r=NR)	Significant intervention effect children's TV viewing, video game use and eating meals in front of TV	Gender<br>Age	NS<br>NS	

					Out-of-school PA by a checklist (r=0.76)				
57. Shilts[65] (2009) (medium=5)	USA 1 middle school n=136 % female=45.0 Mean age=14.0 Drop-out=30.9%	RCT I: 5 weeks, guided goal setting (school curriculum, web-based assessment, workbook with handouts) C: Same intervention without goal setting	1 week before and 1 week after the interventi on (7 weeks post-baseline)	SCT	Dietary and PA behaviour by Centers for Disease Control Youth Risk Behaviour Survey (YRBS) (dietary r=0.73, PA r=0.55)	ITT analysis: No significant effect Non-ITT analysis: Significant effects on dietary behaviour scores	Compliance (to the goal setting)	Significant for dietary behaviour and PA	Stratified by compliance; High compliance > Low compliance
58. Singh[67] (2009) (medium=5)	The Netherlands (DoIT) 18 secondary schools n=1108 %female=53.3 Mean age=12.7 Drop-out=17.6%	RCT I: 8 months, Environmental component (additional physical education classes, changes at school cafeteria) Individual component (education program,11 lessons) C: No intervention	Baseline; 8, 12 and 20 months post-baseline	EnRG DPT TPB HT	Soft drinks and fruit juices consumption High-energy snack consumption, Screen-viewing Active transport	Significant effect on consumption of sugar containing beverages	Gender<br><br>Ethnicity	Significant on screen viewing and soft drink consumption<br>NS	Stratified by gender Screen viewing; Boys > Girls Soft drink consumption; Boys and girls both significant
59. Stevens[68] (2003) (medium=5)	USA (Pathways) 41 schools n=1455 %female=48.3 Mean age=7.5 Drop-out=29.5%	RCT I:3 year trial with a school curriculum, a PA/PE component, a school food service component, and a family component C: No intervention	Baseline; 3 years post-baseline	SLT	PA by Knowledge, attitude and behaviour (KAB) questionnaire (r=NR)	Significant effect on physical activity	Gender<br>Baseline weight status	NS<br>NS	
60. Stock[69] (2007) (low=3)	Canada (Healthy Buddies) 2 elementary schools n=360 %female=55.3	Quasi-experimental I;10 months, peer education by buddy lessons (presentations, games, art activities), PA	Baseline; 10 months post-baseline	-	Healthy behaviour score (includes healthy eating and PA) by Healthy Living Questionnaire (test-retest	Significant effect on health behaviour among 4th through 7th grade	Gender	Significant among students from kindergarten till 3rd grade	Stratified by gender Girls > Boys

		Age range=5-12 Drop-out=35.8%	sessions for buddy pairs (2/ per week) C: No intervention			r=0.70-0.90)				
61.	Vandongen [73] (1995) (low=3)	Australia 30 schools n=1147 %female=51.3 Age range=10-12 Drop-out=24.2%	6-arm RCT I:9 months with 1- Physical fitness, 2-physical fitness+school nutrition, 3-school nutrition, 4-school + home nutrition, 5-home nutrition C:No intervention	Baseline; 9 months post-baseline	-	Total energy, fat, sugar, protein, fibre intake by 2 days dietary recall (r=NR)	NR	Gender	Significant on Fat fibre and sugar intake	Stratified by gender; Fat and fiber intake: Girls > Boys Sugar intake; Boys>Girls

NS= Not significant , **NR**=Not reported, **FV**= Fruit and vegetable, **FJV**= Fruit, juice, vegetable, **SES**=Socio-economic status, **BMI**= Body mass index, **TV**=Television **PA**= physical activity, **LPA**= Light physical activity, **MVPA**=Moderate vigorous physical activity, **VPA**=vigorous physical activity, **PE**=Physical education
SCT=Social cognitive theory, **TM**= Transtheoretical model, **HPM**= Health promotion model, **SEF**=Social Ecological Framework, **TPB**=Theory of planned behaviour, **ELM**=Elaboration likelihood Model, **SLT**=Social learning theory, **HBM**=Health Belief Model, **PBC**=Perceived behavioural control, **TRA**=Theory of reasoned action, **PBT**=Problem behaviour theory, **HBPM**=Health Behaviour Planning Model, **BCT**=Behavioural choice theory, **DPT**=Dual Process Theory, **HT**=Habit Theory, **EnRG**=EnRG framework, **ASE**=Attitude-Social Influence-Efficacy Model, **SM**=Self management, **TTI**= Theory of Triadic Influence, **OLT**= Operant learning theory, **OCT**=Organizational change theory, **DIM**= Diffusion of innovation model, **SAT**= Social Action Theory
